# Genetic architecture of bone quality variation in layer chickens revealed by a genome-wide association study

**DOI:** 10.1038/srep45317

**Published:** 2017-04-06

**Authors:** Jun Guo, Congjiao Sun, Liang Qu, Manman Shen, Taocun Dou, Meng Ma, Kehua Wang, Ning Yang

**Affiliations:** 1 Jiangsu Institute of Poultry Science, Yangzhou, Jiangsu, 225125, China; 2 National Engineering Laboratory for Animal Breeding and MOA Key Laboratory of Animal Genetics and Breeding, College of Animal Science and Technology, China Agricultural University, Beijing, 100193, China

## Abstract

Skeletal problems in layer chickens are gaining attention due to animal welfare and economic losses in the egg industry. The genetic improvement of bone traits has been proposed as a potential solution to these issues; however, genetic architecture is not well understood. We conducted a genome-wide association study (GWAS) on bone quality using a sample of 1534 hens genotyped with a 600 K Chicken Genotyping Array. Using a linear mixed model approach, a novel locus close to *GSG1L*, associated with femur bone mineral density (BMD), was uncovered in this study. In addition, nine SNPs in genes were associated with bone quality. Three of these genes, *RANKL, ADAMTS* and *SOST*, were known to be associated with osteoporosis in humans, which makes them good candidate genes for osteoporosis in chickens. Genomic partitioning analysis supports the fact that common variants contribute to the variations of bone quality. We have identified several strong candidate genes and genomic regions associated with bone traits measured in end-of-lay cage layers, which accounted for 1.3–7.7% of the phenotypic variance. These SNPs could provide the relevant information to help elucidate which genes affect bone quality in chicken.

Osteoporosis in hens is a common disease leading to increased risk to bone fracture, and is especially severe in caged layer populations, yet the underlying genetic architecture is poorly understood. Dissection of the genetic architecture of bone quality is important in both layer production and animal welfare. On the layer production side, the economic losses resulting from osteoporosis are linked to decreased egg production and degraded spent hens due to bone fragmentation during processing^1^,^2^. In terms of animal welfare issues, osteoporosis is identified as the main causal factor for bone fracture in caged layers and is explained as a disturbance in bone metabolism due to eggshell calcium requirement. In the UK, the proportion of hens with broken bones has reached 29%^2^. As a result, there is a need to understand the genetic basis of bone quality in layers, by associating genetic markers with osteoporosis.

Bone breaking strength and BMD are two important parameters to assess the bone quality in layers^3^,^4^. Factors influencing these traits include nutrition, sex, age, exercising, genetics and disease. Genetics as a contributing factor of bone breaking strength has been testified by a number of studies and the heritability of BMD ranged from 0.39 to 0.59 5,6,7. Genetic improvement of bone strength has been carried out in an experimental flock. The genetic gains in this flock suggested that the problem of bone quality could be alleviated by genetic selection^6^. Line diversity on bone strength and BMD has been described, possibly associated with body size or egg production^8^,^9^. Until now, only five studies have mapped quantitative trait loci (QTL) to bone traits in chickens^10^,^11^,^12^,^13^,^14^,^15^. Six QTL affecting femur BMD have been identified on chromosomes 1, 2, 8, 27 and Z. Eight QTL on tibia strength have been identified in chickens. These linkage analyses uncovered not only the genetic loci affecting bone development and strength but also revealed the dominant, maternal and epistasis genetic effects on the bone traits. However, the genetic bases of bone quality-related phenotypes are mainly determined by common variants in humans, each with relatively small effects, rather than the combined effects of several genes^16^. Additionally, QTL mapping studies that applied low density marker panels to a relatively small population, resulted in identified regions with wide confidence intervals. Advances in genotyping technologies make it possible to perform association studies with a large sample size. The genome-wide association study (GWAS) with mixed model and 600 K Chicken Genotyping Panels brings the opportunity to uncover the associated genes with small effects and narrow the genomic regions.

Our aim was to dissect the genetic architecture of bone traits in the chicken using a GWAS approach. Here we obtained the estimate of heritability and genetic correlation using a genomic relationship matrix (GRM) and restricted maximum likelihood (REML) method implemented by GCTA^17^. We also tested the genetic architecture of bone quality variations by partitioning variances according to chromosome and associated single nucleotide polymorphisms (SNPs). In addition, we applied a conditional GWAS to detect independent signals. Together, we performed a GWAS to detect the significant loci and SNPs for bone traits, and provided candidate markers to improve bone strength in hens.

## Results

### Phenotype and heritability analysis

We acquired phenotypic data from a population of approximately 1534 layer-type chickens. The fowls were slaughtered at 72 weeks. Means and standard deviation of bone traits are presented in Tables 1 and 2. The coefficient of variation (CV) of three femur traits ranged from 13.68% to 17.69%. Large CV values obtained from two tibia traits were attributed to greater sample variability. The phenotypic correlation between BMD and breaking strength, two primary interested traits, was close to a high level (with small SD). As shown in Materials and Methods, we used a univariate model to estimate the heritability of bone traits with the genomic relationship matrix. The highest genomic heritability was found in femur weight, followed by femur bone mineral content (BMC) and BMD, with values of 0.43 and 0.33, respectively. Lower heritability was found in two tibia traits. Moreover, bivariate analysis indicated the femur BMD was highly and positively correlated with the tibia breaking strength. The genetic correlation between BMD and breaking strength varied from 0.89 to 0.87 after removal of four overlapping SNPs in GGA1.

### Genome wide association analysis

We tested the association between bone-related phenotypic variants with genotypic polymorphism in the population, controlling for cryptic relatedness and population structure using a mixed model with principal component covariates. Analysis was restricted to the autosomes. The estimated genomic inflation factor for bone traits ranged from 0.98 to 1.04, indicating relatively good consensus between the observed and expected distributions of the P-value.

**Figure 1:**
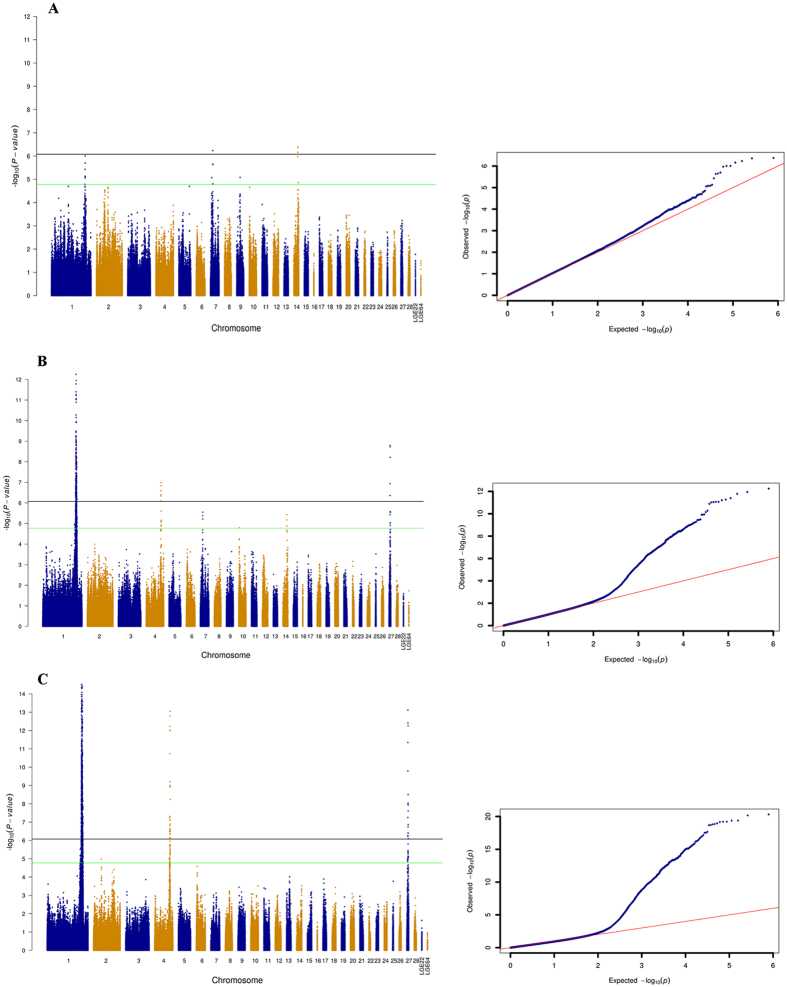
Manhattan and quantile-quantile (QQ) plot displaying the significance signal associated with femur traits. The single-trait analyses on femur BMD (**A**), BMC (**B**) and femur weight (**C**); the horizontal black and green lines indicate the whole-genome significance (P = 8.43 × 10^−7^) and suggestive thresholds (P = 1.69 × 10^−5^), respectively.

**Figure 2:**
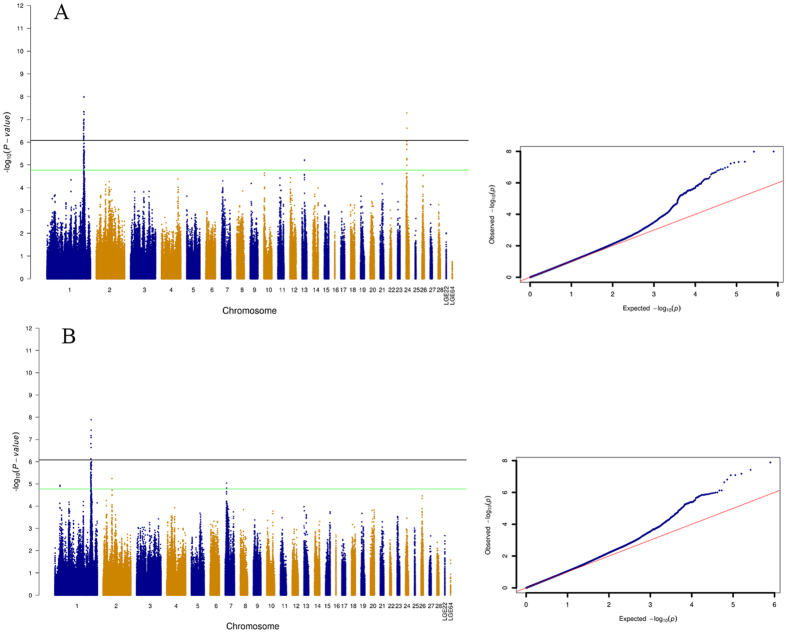
Manhattan and quantile-quantile (QQ) plot on tibia traits. The single-trait analyses on femur breaking strength (**A**) and breaking work (**B**); the horizontal black and green lines indicate the whole-genome significance (P = 8.43 × 10^−7^) and suggestive thresholds (P = 1.69 × 10^−5^), respectively.

The quantile–quantile (QQ) plot revealed that SNPs deviated from the distribution under the null hypothesis, which indicated a strong association between SNPs and BMD (Fig. 1A). Significant genome-wide associations (P = 8.43 × 10^−7^) were examined for four SNPs, of which three were mapped on chromosome 14. The most significant was rs313699988 (P = 4.18 × 10^−7^), which was located at 11.88 Mb. The two significant SNPs on GGA14 were rs314036389 (P = 4.36 × 10^−7^) at 11.87 Mb, and rs313517665 (P = 6.86 × 10^−7^) at 11.57 Mb. Support for the significant association was apparent in a Manhattan plot illustrating that the univariate analysis generated several p-values that exceeded the Bonferroni cutoff (Fig. 1A1). Apart from the region on GGA14 associated with BMD, 13 SNPs were detected for this trait, which were located at the region close to 169 Mb on GGA 1 and 11 Mb on GGA7. The GWAS identified three regions that were significantly associated with femur BMC, and the QQ plot for BMC supported the results shown in the Manhattan plot (Fig. 1B). Three regions were located on GGA 1, 4 and 27. For femur weight, a peak of genome-wide significant SNP effects was found on GGA 1 (P = 4.75 × 10–21). Almost all significant associated loci located in this region spanning from 164.80 Mb to 173.85 Mb on GGA 1. Out of this region, 28 SNPs on GGA 4 built up an associated block ranged from 73.48 Mb to 76.94 Mb, and a region located between 3.03 Mb and 3.63 Mb on GGA 27 showed significant at the genome-wide level. The distribution of significant SNPs related to tibia traits was shown in Fig. 2.

To uncover the relevance of genotyping data to the bone breaking traits, we examined their associations with measurements in 3-point bending test. QQ plots were used as a visual method to display the difference in expected and observed p-values. Here, QQ and Manhattan plots were constructed but they only detected several SNPs were significantly associated with bone breaking strength. The five SNPs were located at a genomic region between 167.83 Mb and 169.41 Mb on GGA1. The genomic region on GGA24 contains two SNPs that were associated with the tibia breaking strength at the genome-wide level. Apart from tibia breaking strength, breaking work was significantly associated with the genomic region on GGA1.

### Conditional analysis

**Figure 3:**
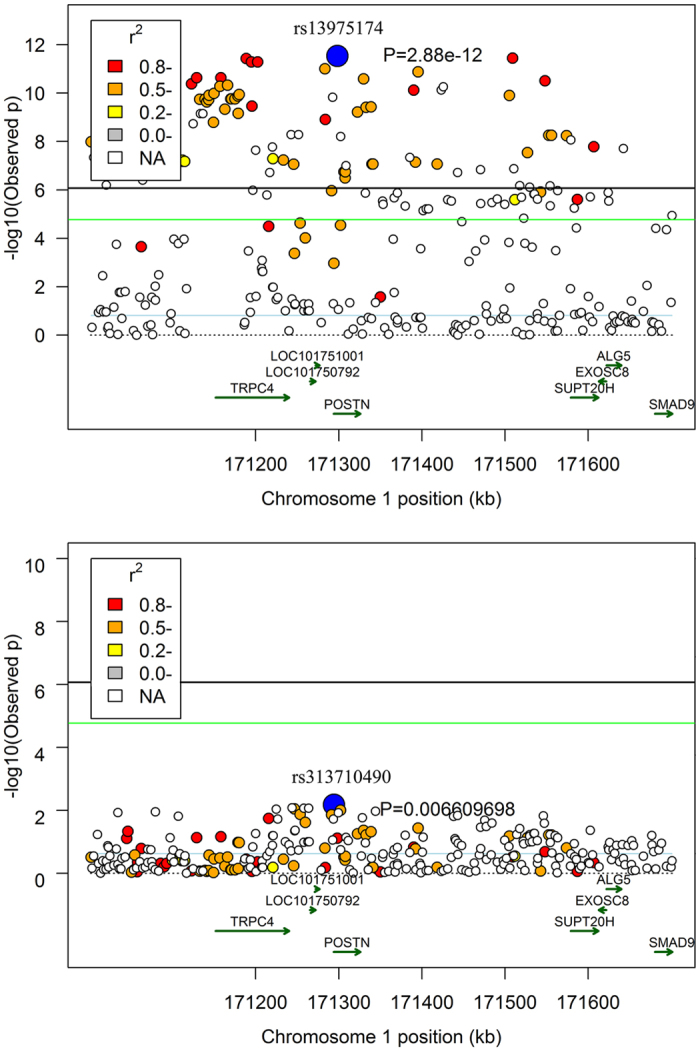
Regional association plot of the primary signal (rs13975174) associated with femur weight at GGA1. For each plot, the −log_10_ (observed P-values) of SNPs (y-axis) are presented according to their chromosomal positions (x-axis). The blue line indicates the genome-wide significance level (8.43 × 10^−7^), and the red line the predicted level. The primary SNP are denoted by large blue circles. SNPs are represented by colored circles according to the target SNP with which they were in strongest LD (*r*^2^ > 0.4). The upper part of the figure shows the association results for BMC before conditional analysis on rs13975174. In the lower part of the figure, the P-value of corresponding SNPs fell below the predicted threshold.

Stepwise conditional analysis was conducted to examine whether there are cryptic, independently associated SNPs that might act as potential causal variants. We performed conditional single-marker association analyses on rs315077363 to uncover the signals potentially masked by the strong signals in GGA1. The P-values of previous significant SNPs for BMC on GGA1 were lower than the suggestive level, whereas an SNP at 169.4 Mb on GGA1 was identified as the significant marker, which were located in the intron of the *INTS6* gene. For femur weight, a SNP located in the intron of *POSTN* gene on GGA1 was also identified as a significantly associated marker. Moreover, no significant association was found on GGA1 after conditional analysis of two additional variants. In our conditional analyses, only SNPs on GGA1 were selected, and no additional signals were detected except for this region. The results on region-specific and conditional analysis for the BMC are presented in Fig. 3 and Figure S1.

### Dissection on phenotypic variance by allelic variants

Considering the complexity of the bone metabolism, we looked for genes clearly known to be involved in the regulation of osteoclast or osteoblast function in close vicinity of the associated SNP and identified a total of 10 candidate genes or gene families (Table 3). Particularly, a genomic region located at 165–171 Mb on GGA1 had some genetic overlap between five bone traits in this study. In this region, a missense mutation (rs312550725: T222M) in the *SERPINE3* gene was identified as a potential causative variant for breaking strength. Although the P-value of the rs315096388 marker did not reach a significant threshold for BMD (P-value = 9.85 × 10^−7^), we hypothesized that this marker had a potential diagnosis on osteoporosis depend on the allelic frequency and the degree of linkage disequilibrium between the SNPs and *ADAMTS15* which was the candidate gene with osteoporosis and osteoporotic fractures^18^.

The REML method implemented in the GCTA program allowed us to estimate the genetic variances through the SNP-based genetic relationship matrix. Bone phenotypic variance explained by candidate gene polymorphisms are presented in Table 3. The largest estimate was obtained from femur weight, with the proportion reaching 0.077. For BMD and breaking strength, two important parameters on osteoporosis diagnosis, the positive beta values presented in Table 3 indicate that the minor allele at the candidate gene was associated with an increase in corresponding measurements^19^.

### Partitioning of the genomic variants by chromosomes

**Figure 4:**
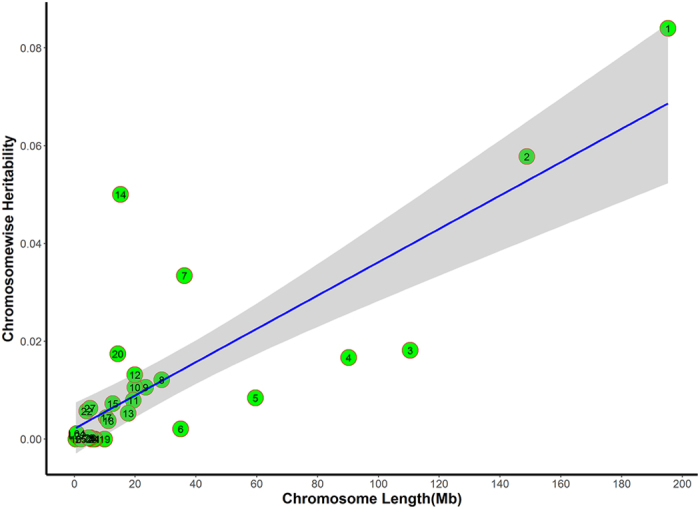
Heritability of femoral BMD by chromosome. Estimates of chromosome-wise heritability on BMD are drawn against the chromosome length (x-axis). The blue line represents heritability regressed on chromosome length. Grey area around the blue line is the 95% confidence level interval for prediction from the linear model. Chromosomes 7, 14, and 20 fell outside of the 95% confidence interval, indicating that these chromosomes could explain more heritability than expected according to chromosome length.

**Figure 5:**
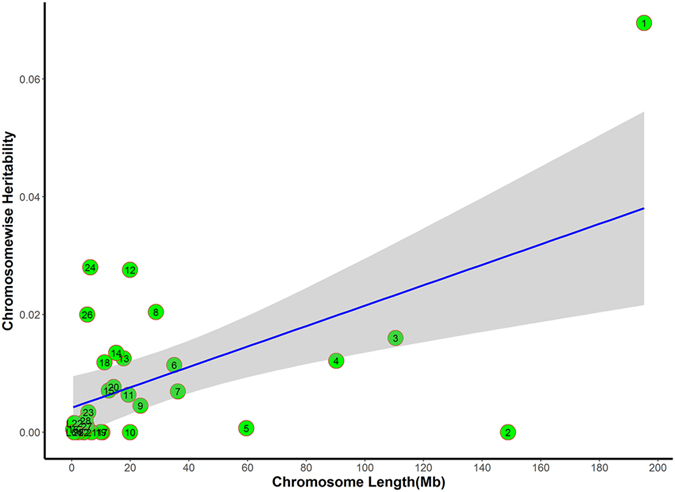
Heritability of tibia breaking strength by chromosome. Chromosomes 1, 8, 12–14, 18, 24 and 26 lie outside the 95% confidence interval, indicating that these chromosomes could explain more additive variances than expected according to chromosome length.

The phenotypic variance explained by each chromosome is shown in Figs 4 and 5. Heritability obtained from the joint analysis of variance explained by each chromosome were linearly related with the chromosome lengths for BMD and breaking strength (Adjusted R^2^ = 0.64 and 0.57, respectively). These results agreed with the infinitesimal model theory, that is, common variants throughout the genome dissected the total variances. In total, 28 autosomes and two linkage groups account for 37.73% of BMD variance and 28.47% of breaking strength variance. These results were not different than the univariate heritability estimates (Table 3). The contribution of particular chromosomes differed from each other. Three chromosomes, GGA1, GGA2 and GGA14, explained more than 5.00% of BMD variance, indicating more genetic contributions than other chromosome segments. The contribution of GGA2 accounted for a large fraction of BMD variance, although association analysis with a mixed model did not identify significantly associated SNPs on this chromosome.

## Discussion

Previous genome-wide scanning for markers of the bone quality in layer chickens were limited to the QTL linkage analysis, which often had large confidence intervals to compromise precise localization on causal variants. In this study, we integrated a high-density microarray panel, robust phenotypic assessments and statistical analysis strategies with an aim to dissect the genetic architecture of the bone quality in chickens. We found a substantial overlap in chromosome 1 that contributed to the differences in the bone traits in a crossbred layer population. This region was similar to a reported QTL for tibia BMD on chromosome 1 in a broiler × layer cross^12^. Dunn *et al*.^14^ reported significant QTL for tibia breaking strength, with the location of the QTL close to the region identified in this study^14^. In another study, where Jungle fowl and White Leghorns were used to set up the resource population, a QTL related to noncortical BMD was reported, with the confidence intervals close to the region identified in this study^11^. Other positive SNPs on bone breaking strength were also similar to those reported for QTL in a Leghorn and Fayoumi cross^10^. This indicates that GWAS successfully identified SNPs with genetic variation attributable to bone quality in chicken.

Our results provide support for the hypothesis that common variants primarily contribute to the difference in bone quality. In this population, we found common variants accounted for 35%, 46% and 26% of phenotypic variation for BMD, BMC and tibia breaking strength, respectively. Heritability measured with genomic relationship matrix was similar to that done with pedigree matrix^6^,^20^. We found variance explained by chromosomes was in approximate proportion to each chromosome length. Although this reflected that many SNPs with small effects influenced bone quality traits, the estimates were not perfect due to rare indels and structural variants that were not considered in this study. The greatest heritability for BMD was mapped on chromosome 1. A significant effect on bone quality was associated with a region of 165–171 Mb on GGA1. In this region, four genes (*RANK, SERPINE3, INTS6* and *POSTN*) were identified in the NCBI genomic biology database. These genes might have an important effect on bone quality.

*RANK*, also known as *TNFS11*, was a strong candidate gene for BMC based on the close link with the marker rs312784357. The RANK–RANKL–OPG axis is involved in osteoclast cell differentiation as well as its activation^21^. A clinical reagent containing the monoclonal anti-RANKL antibody, which could inhibit the osteoclastogenesis and bone resorption of mature osteoclasts, was used to treat osteoporosis^22^. In chicken, the osteoclast-like multinucleated cells induced by the recombinant ch*RANKL* protein were observed in a dose-dependent manner^23^. A second hit for bone traits on GGA1 was *SERPINE3*, which is a member of a gene family of the serine proteinase inhibitor (serpin) known to participate in extracellular matrix regulation^24^. *SerpinF1*-deficient patients display susceptibility to fractures after minimal trauma^25^. In this study, two important assessments, BMD and breaking strength, were associated with this candidate gene. A third hit for bone quality on GGA1 lay in the intron of the integrator complex subunit 6 (*INTS6*), which contained significant SNP effecting BMC and tibia breaking work. Two studies are in support of *INTS* having an effect on bone quality. In a study aimed to scan differentially expressed genes between osteoporosis patients and controls, *INTS* is present in the list of down-regulated genes^26^. In another study on differently expression genes, *INTS* was identified with osteoblast differentiation and proliferation^27^. The fourth hit for bone traits on GGA1 was *POSTN*, which was an extracellular matrix protein that had an essential role as a regulator of osteoblast differentiation and bone formation. Further studies suggested that the periostine-sclerostine-Wnt/β-catenin pathway plays a beneficial role in bone metabolism, with up-regulation in osteoblastic formation, and activation of the osteocyte^28^,^29^,^30^.

Studies in mice and rats suggestedthat BMD traits are positively correlated with bone breakage^31^, but there are few heritability estimates for breaking strength. In this study, we estimated the genetic parameters on the tibia breaking strength with a genomic relationship matrix. We provided evidence that there was a narrow region on GGA1 involved in the regulation of both BMD and breaking strength, which accounted for the relatively high genetic correlations between these traits. Another potential locus associated with tibia breaking strength was identified on chromosome 24. Although the P value for the associated SNP (rs315096388) with the breaking strength was slightly higher than the significant threshold of 8.43e-7, we thought that a weak association existed between this signal with breaking strength, but the results require further studies. This proposed effect on breaking strength associated with the rs315096388 marker explains ~1.2% of the phenotypic variance. The rs315096388 SNP is located in the 3′-untranslated region of the *ADAMTS15* gene. In humans, the *ADAMTS* family plays a role as a regulator of bone development and remodeling. *ADAMTS* was verified as a genetic susceptibility loci for osteoporotic fracture in different ethnic populations^18^.

In the current study, we found three SNPs on chromosome 14 were significant on the genome-wise level. Two of these SNPs were located in the downstream region of the *GSG1L* gene, which encodes a core subunit of an AMPA receptor, one was located between an uncharacterized gene and the interleukin 20 receptor gene. Based on current knowledge of gene ontology, it seems that the femur BMD associates with SNPs close to the *GSG1L* gene. Recently, several research groups propose that the glutamate receptor played an important role in the regulation of bone growth and remodeling^32^,^33^. An immunohistochemical study has demonstrated that *AMPAR* was constitutively expressed in primary rat osteoblasts^33^. The product of the *GSG1L* gene suppressed the calcium-permeability of the receptor by reduced single-channel conductance^34^. The maturation process of osteoblasts requires the activation of *AMPAR*^35^. Bone is a highly innervated tissue consisting of nerve fibers. Bone remodeling and metabolism is controlled by numerous regulatory mechanisms. We infer that the *GSG1L* gene is a loci for osteoporosis susceptibility with a view to developing our understanding of bone biology.

In addition to the significant loci on chromosome 1 and 4, we also identified the rs316237751 SNP associated with BMC mapping to chromosome 27. This rs316237751 marker with relatively low minor-allele frequency (MAF) (8.7%) was close to the SOST gene. Prior to this study, mutations in the *SOST* gene were identified as a candidate gene of BMD variations in humans^36^,^37^. *SOST*, a protein secreted by osteocytes, could down-regulate osteoblastic bone formation. A loss of function mutation in the *SOST* gene led to generalized osteosclerosis with high BMD and strong bones^38^. As discussed in the previous part on POSTN, SOST involved in the wnt/β-catenin signal pathway. *SOST* directly interacts with Low Density Lipoprotein Receptor-Related Protein 5 and Low Density Lipoprotein Receptor-Related Protein 6 and antagonizes Wnt signaling both *in vitro* and *in vivo*^39^,^40^. In this context, the findings from association analysis highlighted the role of the *SOST* gene during the regulation of bone quality, and endowed this gene with both promising and encouraging applications in layer improvement.

In summary, significant genetic variation for bone quality was identified on chromosomes 1, 4, 14, 24 and 27 using mixed model analysis. Several promising candidate genes involved in bone development were mapped to narrow regions. However, this requires further validation to overcome the relatively limited power due to long range linkage disequilibrium. We thought the genetic variance on bone breaking strength and BMD in chicken could be explained by common variants. The values of genetic parameters based on genomic relationship matrix had not been reported before for chicken, and these parameters are important for the selection of bone quality. The identification of such associated SNPs represented a key pace forward in dissecting the genetic basis of bone quality in chicken and could help to alleviate osteoporosis in layer chickens.

## Materials and Methods

### Ethical statement

The fowls were reared and slaughtered in compliance with the guidelines for the care and use of experimental animals approved by the Ministry of Agriculture of China. Study design was approved by the local Institutional Ethic Committee of Jiangsu Poultry Science Institute.

### Animals

The population used in this study were two layer lines established from a crossbred flock in an F2 design as previously described^41^. Briefly, a Chinese indigenous breed with blue eggs was selected for reciprocal mating with a White leghorn flock. The pedigree structure contained 12 sires and 213 dams from the parent generation, 49 males and 639 females from the F_1_ generation, and 1534 hens from the F_2_ generation. Among these animals, only F_2_ birds were measured for phenotypic data of interest. All chicks were housed in temperature- and light-controlled rooms for 42 days. In the first week after hatching, the chicks were given artificial illumination throughout the night. The photoperiod was then decreased by 1 h/week until 9 h of light was provided. The fowls were housed under natural light until transfer to single-hen cages at 16 weeks of age. The light treatment was then gradually increased by 1 h per week until 16 h of light was provided. The laying mash contained 16.16% crude protein, 10.64 MJ kg^−1^ metabolisable energy, 3.4% calcium and 0.52% total phosphorus.

### Trait measurements

Fowls were killed at 72 weeks for bone measurements. The right femur and tibia were dissected from the carcass. Muscles and connective tissue were carefully removed from the bone with a scalpel. The weight of the femur was recorded. Measurements of breaking strength and work of the right tibiotarsus were carried out on an TMS-Pro food testing instrument (Food Technology Corporation, VA, USA). BMD and BMC of the femur were determined by dual energy X-ray absorptiometry using the Discovery DXA system (Hologic, Inc. Bedford, MA, USA).

### Genotyping

DNA was extracted from blood tissue with a standard protocol. Genotyping analyses of 1534 samples from F2 generation were performed with a Affymetrix^®^ Axiom^®^ 600 K Chicken Genotyping Array (Affymetrix, Inc., Santa Clara, CA, USA). More details on the genotyping array are described by Kranis *et al*.^42^. The quality of array data was evaluated with Affymetrix Power Tools and PLINK software^43^. SNPs were excluded if they had a minor-allele frequency <1%. SNPs that deviated from the Hardy–Weinberg equilibrium (HWE) (P value < 1e−6) were removed. SNPs on sex chromosomes were removed. Samples with call rates <95% were removed. This led to the removal of 22 samples from the data set. Phasing analyses were performed with Beagle software (version 4.0)^44^. Finally, 435,867 autosomal SNPs and 1512 samples passed the quality control.

### Association analysis

Before the association test, an independent SNPs set was established by PLINK, with a window size of 25 SNPs, a step of five SNPs, and an r^2^ threshold of 0.2. Then, we obtained principal components (PCs) using linkage equilibrium SNPs. The top five PCs were assigned as covariates in a linear mixed model. We selected a simpleM method to infer the thresholds for genome-wide significance and proposed associations. In this study, the effective number of independent tests was 59,308, thus the genome-wide predicted and significant P-values were 1.69e-5 and 8.43e-7, respectively. We searched for candidate genes that were closest to associated SNPs in Ensembl (http://asia.ensembl.org) and NCBI (http://www.ncbi.nlm.nih.gov).

A standard linear mixed model, implemented in GEMMA, was used to correct the stratification of the study population^19^. The statistical model used for the association test was presented by the matrix expression:


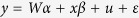


where, y is the vector of phenotypic values for all fowls, W is the incidence matrices of covariates effect including the top five PCs, α is the vector of corresponding coefficients including the intercept, x is the vector of marker genotypes, β is the corresponding effect size of the marker. u is the vector of random effects, with u~(0, GRM 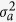), where 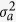 is the genetic variance and GRM represents the genomic relationship matrix. Here, GRM was calculated with the GCTA package and was used to replace A matrice^17^. ε is the vector of random residuals with ε~N (0, I *σ*^2^), where *σ*^2^ is the residual variance component. The applied statistical model was as described previously for single-marker GWAS and included egg production up to 72 weeks as a covariate for the analyses of BMD, BMC, breaking strength and work. Wald test statistic was used as standard to select SNPs associated with bone traits. The genomic inflation factor was calculated by the R package GenABEL^45^. The Manhattan plots were generated with R as described^41^. QQ plots were used to analyze the extent to which the observed distribution of the test statistic followed the expected distribution. The QQ plots were obtained with R package gap^46^.

### Conditional analysis

Conditional analysis was conducted to examine the potential associated SNPs that might be masked by a strong signal. Briefly, the initial screen was tested with the strongest SNP covariate. Then, association analysis conditioning on the selected SNP(s) was implemented to search for the top SNPs iteratively one by one via a stepwise model selection procedure until no SNP had a conditional P-value that passed the significance level. GWAS does not distinguish a genuine causal locus from those statistically significant loci within a strong linkage disequilibrium (LD) region. Therefore, in order to characterize potential candidate genes responsible for a trait, we first conducted an LD analysis and inferred the haplotype blocks containing peak SNPs by Haploview v4.2^47^. A block was derived using the solid spine algorithm, and defined by the first and last SNPs in a region that were in strong LD (D′ ≥ 0.8) with all intermediate SNPs. Regional association plots were generated using gap packages^46^.

### Heritability

To investigate a role for additive genetic contributions to osteoporosis susceptibility, we applied a restricted maximum likelihood method for estimating heritability based on the genomic relationship matrix as implemented in the GCTA package. It estimated the genetic variance explained by chromosome and individual SNPs. GCTA allowed the relationships among individuals to be replaced by GRM, and also permitted principal component analysis (PCA) eigenvectors embedding as covariates to capture variance due to population structure. In this analysis, the first five PCAs were included as covariates, in order to adjust the structure of the stratified population and the cryptic relatedness.

## Additional Information

**How to cite this article**: Guo, J. *et al*. Genetic architecture of bone quality variation in layer chickens revealed by a genome-wide association study. *Sci. Rep.* **7**, 45317; doi: 10.1038/srep45317 (2017).

**Publisher's note:** Springer Nature remains neutral with regard to jurisdictional claims in published maps and institutional affiliations.

## Supplementary Material

Supplementary Information

## Figures and Tables

**Table 1:** Summary of bone traits in the population.

Traits	Sample size	Minimum	Maximum	Mean	SD
Femur BMD g/cm^2^	1406	0.200	0.580	0.329	0.045
Femur BMC g	1406	1.630	5.310	2.996	0.530
Femur weight g	1409	3.100	9.300	6.180	0.895
Tibia breaking strength N	1397	45.000	394.200	189.054	55.397
Tibia breaking work N·mm	1393	15.100	539.200	139.828	77.273

**Table 2:** Heritability, genetic and phenotypic correlations in bone traits^1^.

	Femur BMD	Femur BMC	Femur weight	Tibia breaking strength	Tibia breaking work
Femur BMD	**0**.**35 ± 0**.**05**	0.86 ± 0.01	0.52 ± 0.02	0.72 ± 0.01	0.52 ± 0.02
Femur BMC	0.83 ± 0.03	**0**.**46 ± 0**.**04**	0.76 ± 0.01	0.49 ± 0.02	0.68 ± 0.02
Femur weight	0.48 ± 0.07	0.86 ± 0.03	**0**.**62 ± 0**.**04**	0.57 ± 0.07	0.64 ± 0.09
Tibia breaking strength	0.89 ± 0.04	0.80 ± 0.05	0.58 ± 0.07	**0**.**26 ± 0**.**04**	0.72 ± 0.01
Tibia breaking work	0.76 ± 0.09	0.82 ± 0.08	0.69 ± 0.09	0.92 ± 0.05	**0**.**14 ± 0**.**04**

Corresponding SD behind the mean heritabilities on diagonal (bold), phenotypic correlations above diagonal, genetic correlations below diagonal.

**Table 3:** Loci associated with bone traits in crossbred layer population.

Chromosome	SNP	Physical position^a^	Minor/major Allele	Minor Allelic Frequency	Candidate gene	SNP location	Proportion phenotypic variance	Beta^b^	SE^c^	P Value	Trait
1	rs312784357	165885243	A/G	0.480	RANKL	intergenic	0.050	-0.298	0.057	3.09E-07	BMC
1	rs312550725	169381724	T/C	0.405	SERPINE3	missense	0.013	0.226	0.047	4.71E-08	Breaking strength
1	rs317281616	169400244	G/A	0.307	INTS6	intron	0.030/0.056	0.275/0.345	0.049/0.055	1.29E-08/5.86E-10	Breaking work/BMC
1	rs13975174	171298203	A/G	0.370	POSTN	intron	0.077	−0.293	0.050	2.88E-12	Femoral weight
4	rs14491507	76073771	A/G	0.052	TAPT1	intergenic	0.024	0.479	0.090	1.11E-07	BMC
4	rs15620367	76186390	A/G	0.130	FGFBP1	intron	0.023	0.333	0.066	5.51E-07	Femoral weight
14	rs313699988	11876668	T/C	0.126	GSG1L	intergenic	0.023	0.315	0.062	4.18E-07	BMD
24	rs315096388	1684374	T/C	0.177	ADAMTS15	intergenic	0.014	0.215	0.051	2.43E-07	Breaking strength
27	rs316237751	3200813	G/C	0.087	SOST	intergenic	0.031	0.430	0.071	1.59E-09	BMC
27	rs316723909	3464323	G/A	0.087	IGF2BP1	intergenic	0.041	0.500	0.072	5.01E-12	Femoral weight

^a^Physical position is bp location on chromosome for NCBI Gallus_gallus-4.0. ^b^Estimates presented for allelic substitution effect per copy of the effect allele (EA) under an additive model, expressed in SD unit/allele.

^c^SE is the standard error of the beta.
