# Supplementary material for: Genetic architecture of bone quality variation in layer chickens revealed by a genome-wide association study
Source: Sci Rep. 2017 Apr 6;7:45317. doi: 10.1038/srep45317 (PMC5382839; doi:10.1038/srep45317)
Supplement: Supplementary Information [file srep45317-s1.pdf]

Title: Genetic architecture of bone quality variation in layer chickens revealed by a genome-wide association study

Author:

GUO Jun[1]
Phone number:13615250109
Email:guojun.yz@gmail.com

SUN Congjiao[2]
Phone number:15910707895
Email: cjsun@cau.edu.cn

QU Liang[1]
Phone number:15952760690
Email:quliang19820603@163.com

SHEN Manman[1]
Phone number:15952730756
Email:shenman2005@163.com

DOU Taocun[1]
Phone number:15995162952
Email: yzdtc@126.com

MA Meng[1]
Phone number:18796626219
Email:779701121@qq.com

WANG Kehua[1*]
Phone number:13805276606
Email: kehua_wang@126.com

YANG Ning[2*]
Telephone number: +86-10-6273 1351
Email:nyang@cau.edu.cn

[1]Jiangsu Institute of Poultry Science, Yangzhou, Jiangsu, 225125, China
[2]National Engineering Laboratory for Animal Breeding and MOA Key Laboratory of Animal Genetics and Breeding, College of Animal Science and Technology, China Agricultural University, Beijing, 100193, China

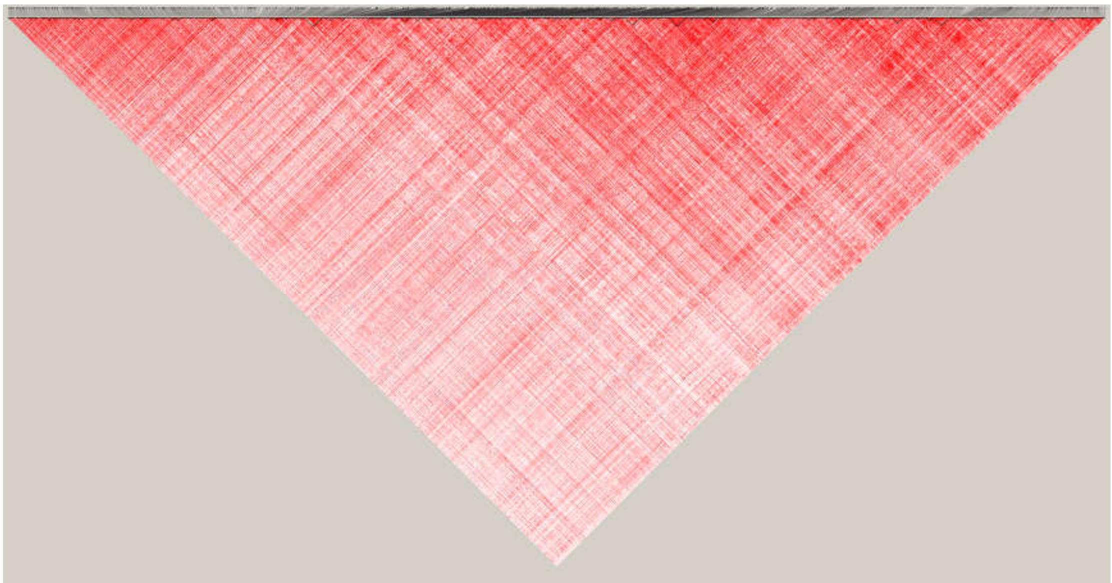

Figure S1 linkage disequilibrium around significant SNPs on GGA1

Haplotype block (166.5-174.5 Mb) containing all significant SNPs from the genome-wide association study. There were four candidate genes in this region, i.e. *RNKL*, *SERPINE3*, *INTS6* and *POSTN*.
